# Quantitative assessment of carotid plaque surface irregularities and correlation to cerebrovascular symptoms

**DOI:** 10.1186/1476-7120-11-38

**Published:** 2013-11-06

**Authors:** Baris Kanber, Timothy C Hartshorne, Mark A Horsfield, A Ross Naylor, Thompson G Robinson, Kumar V Ramnarine

**Affiliations:** 1 Department of Cardiovascular Sciences, University of Leicester, Leicester, England, UK; 2 Department of Surgery, University Hospitals of Leicester NHS Trust, Leicester, England, UK; 3 NIHR Biomedical Research Unit for Cardiovascular Sciences, University of Leicester, Leicester, England, UK; 4 Department of Medical Physics, University Hospitals of Leicester NHS Trust, Sandringham Building, Leicester Royal Infirmary, Infirmary Square, LE1 5WW, Leicester, England, UK

**Keywords:** Carotid plaque, Surface irregularities, Surface irregularity index, Cerebrovascular symptoms

## Abstract

**Background:**

The purpose of this study was to determine whether surface irregularities measured from ultrasound images of carotid artery plaques and quantified using a novel method, correlate with the presence of ipsilateral hemispheric cerebrovascular symptoms.

**Methods:**

A plaque surface irregularity index (SII) was measured in 47 carotid artery plaques (32 subjects, stenosis range 10% -95%, 49% symptomatic) using ultrasound image sequences spanning several cardiac cycles. The differences in the distribution of SII in plaques with ipsilateral hemispheric symptoms versus those without symptoms and the correlation between the SII of plaques and the degrees of stenosis of the corresponding arteries were assessed. Diagnostic performance of plaque SII was evaluated on its own and in combination with the degree of stenosis.

**Results:**

The mean SII was significantly greater for plaques with ipsilateral hemispheric symptoms (1.89 radians/mm) than for asymptomatic plaques (1.67 radians/mm, p = 0.03). There was no statistically significant association between the SII and the degree of stenosis (p = 0.30). SII predicted the presence of cerebrovascular symptoms with an accuracy of 66% (sensitivity 65%, specificity 67%) on its own and with an accuracy of 83% (sensitivity 96%, specificity 71%) in combination with the degree of stenosis.

**Conclusions:**

Quantitative assessment of carotid plaque surface irregularities using a novel SII parameter correlates with the presence ipsilateral hemispheric cerebrovascular symptoms and may increase diagnostic performance beyond that provided by the degree of stenosis.

## Background

There is growing interest in using ultrasound images of the carotid artery to assess plaque surface irregularities and use this as a surrogate marker of carotid plaque ulceration and vulnerability. Previous studies have investigated plaque surface irregularities using qualitative classification schemes such as smooth vs. irregular or by using specific criteria for classifying ulceration [1-5]. Surface structure determined from ultrasound images has been found to correlate, to some extent, with surface structure found on angiography [6,7], intra-plaque haemorrhage [8], CT/MRI-determined cerebral infarctions [9-12] and the incidence and presence of cerebrovascular events and symptoms [6,8,9,12-16]. Ultrasound-determined surface structure agreed with that found on surgical/autopsy specimens with varying degrees of success [1,3,5,17-26]. A large, prospective study found that the unadjusted, cumulative, 5-year risk of ischaemic stroke was 8.5% when irregular plaques were seen on ultrasound, compared to 1.3% and 3.0% for no plaque and smooth plaques, respectively [27].

Quantitative assessments of plaque surface irregularities may have benefits over qualitative assessments, since they should be more operator-independent. Yet, even with quantitative analyses, manual delineation of the plaque-arterial lumen boundary on ultrasound images introduces some subjectivity into the process and is less likely to capture small defects on the plaque surface. There have been only a few attempts to quantify the surface irregularities of carotid artery plaques. Tegos et al. [28] quantified surface irregularities by calculating the bending energy of the plaque surface. However, plaque surfaces were manually outlined by the operator, and the authors obtained similar bending energies for symptomatic and asymptomatic plaques. More recent studies quantified plaque surface irregularities by measuring the principal curvatures of plaque surfaces in 3 dimensions [29,30]; however, the underlying 3-dimensional ultrasound techniques are still under development, and more difficult to implement in the vascular clinic compared to 2-dimensional techniques.

We hypothesized that an objective, quantitative measurement of carotid plaque surface irregularities using 2-dimensional, cross-sectional ultrasound imaging would correlate with the presence of ipsilateral hemispheric symptoms. This study defined a novel surface irregularity index (SII) and investigated whether it enhances diagnostic performance compared to the degree of stenosis of the carotid artery alone.

## Methods

32 consecutive patients (20 males and 12 females) who attended the University Hospitals of Leicester NHS Trust’s Rapid Access Transient Ischaemic Attack (TIA) clinic were recruited. The study was approved by the National Research Ethics Service (NRES) Committee East Midlands - Northampton (reference 11/EM/0249), followed institutional guidelines, and each patient gave informed consent before participating in the study. Patients who did not have carotid stenoses were excluded from the study. In total, surface irregularity indices of 47 carotid artery plaques (stenosis range 10% -95%) were measured. Plaques were classified as either having caused ipsilateral hemispheric cerebrovascular symptoms (i.e. symptomatic) or asymptomatic following specialist medical review. Symptoms included aphasia, transient monocular blindness and hemimotor/sensory symptoms consistent with transient ischaemic attack or stroke.

### Data acquisition

Longitudinal cross-sections of the carotid plaque were acquired by experienced sonographers using a Philips iU22 ultrasound scanner (Philips Healthcare, Eindhoven, The Netherlands) with an L9-3 probe. B-Mode (greyscale) and Colour Doppler image sequences were recorded as DICOM files over an average of 5 cardiac cycles (mean frame rate was 32 frames per second) using the vascular carotid preset on the scanner (Vasc Car preset, persistence low, XRES and SONOCT on). Colour Doppler image sequences were used as a qualitative aid to identifying the location and extent of the plaques, and for qualitative assessments, while the greyscale data were used for the quantitative analyses of plaque surface irregularities.

### Analysis

Quantitative analyses were carried out using MATLAB version 7.14, release 2012a (MathWorks, Natick, Massachusetts, USA) and employed a novel technique to track plaques throughout ultrasound image sequences [31]. We measured plaque surface irregularities using a novel surface irregularity index which was calculated by computationally summing the angular deviations from a straight line, of the luminal plaque surface, and dividing this by the length of the plaque surface. The measurements were made without *a priori* knowledge of the patient symptomatic status. The surface irregularity index was also combined with the degree of stenosis of the corresponding artery by taking their product, resulting in a combined risk indicator. Degrees of stenosis were measured using criteria consistent with the NASCET method utilizing blood flow velocities in conjunction with the B-Mode and colour flow imaging [32-34] and plaque SII measurements were averaged across all image frames. As Doppler velocity measurements are not able to reliably discriminate degrees of stenosis below 50%, we used B-Mode diameter measurements and colour flow imaging to grade the degree of stenosis into deciles for minor stenoses. We assessed the reproducibility of our surface irregularity measurements by calculating the intra-observer and inter-frame variabilities. Intra-observer variabilities were determined by measuring the surface irregularity indices of nine selected plaques five times using the same carotid file-video for each plaque, respectively. The nine plaques were selected from the available dataset to give a wide range of stenosis severity and plaque echogenicity for reproducibility analysis. Inter-frame variabilities, on the other hand, were assessed for all the plaques included in the study, to give a measure of the magnitude of variations seen in the surface irregularity indices across image frames. A qualitative assessment of plaque surface irregularities was also performed by an experienced vascular scientist, off-line and blinded to patient clinical history, classifying plaque surfaces as either smooth or irregular using the greyscale and Colour Doppler images as a guide.

### Statistical analysis

Statistical analyses were carried out using SPSS version 20 (IBM Corporation, Armonk, New York, USA). The non-parametric Mann–Whitney U-test was used to determine whether the surface irregularity indices differed significantly between the symptomatic and asymptomatic plaque groups and those plaques qualitatively classified as having an irregular or smooth surface. Kendall’s tau was used to establish whether the SII, the degree of stenosis, and the plaque area could be regarded as statistically independent and Receiver Operating Characteristic (ROC) curves were used to investigate the diagnostic performance of the plaque SII on its own and in combination with the degree of stenosis. The correlation between the symptomatic and asymptomatic plaque groups and the qualitative plaque surface assessment was performed using Pearson’s χ^2^.

## Results

Twenty-four of the 47 plaques investigated were found to be free from symptoms, while the remaining 23 were found to be symptomatic following expert specialist stroke physician assessment. The mean age of the symptomatic patients was 75.3 years compared with 77.8 years for the asymptomatic (p > 0.05, Mann–Whitney test). None of the patient characteristics sex (20 males), current or past tobacco smoking (63%), hypertension (63%), hypercholesterolaemia (53%), diabetes mellitus (53%), ischaemic heart disease (38%), family history of stroke (34%), previous TIA/stroke (44%), alcohol consumption (28%) and peripheral vascular disease (13%) had a statistically significant relationship to the presence of symptoms (p > 0.05 for all, Pearson’s χ^2^).

**Figure 1:**
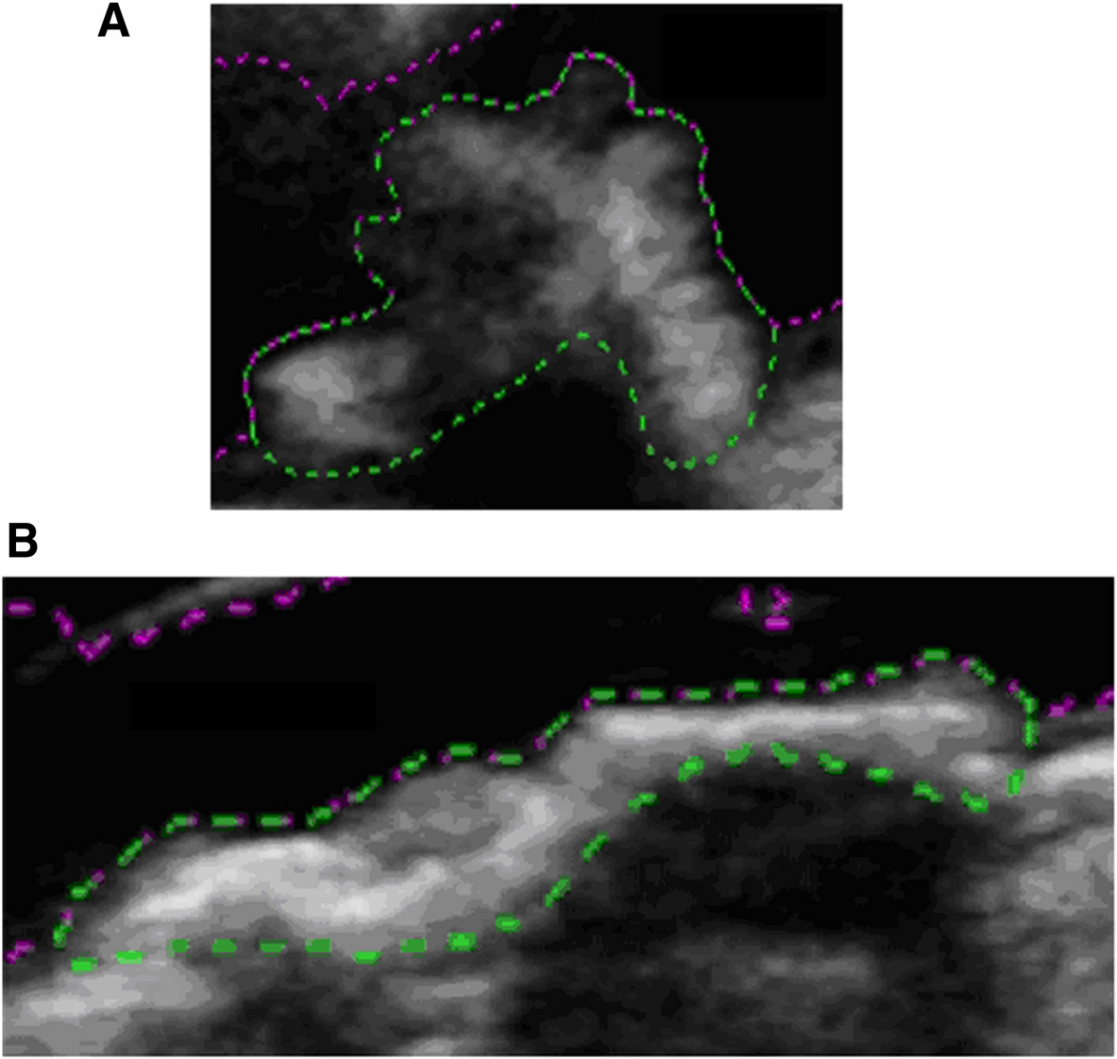
**Two plaques of markedly different surface irregularity indices: (a)** a symptomatic plaque with an SII of 2.25 radians/mm; and **(b)** an asymptomatic plaque with an SII of 1.57 radians/mm. The plaque surface is the boundary between the plaque and the arterial lumen (where the pink and green dashed lines overlap). **(a)** is also a plaque qualitatively classified as having an irregular surface, while **(b)** is a plaque qualitatively classified as having a smooth surface.

**Figure 2:**
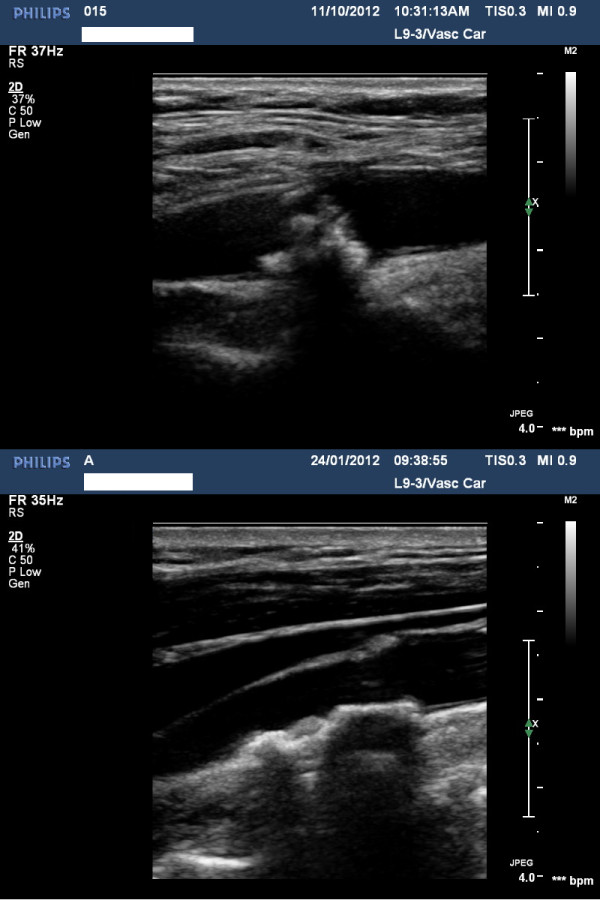
**Full-size ultrasound images corresponding to the close-up plaque views shown in Figure **1**.** (top row) the symptomatic plaque, (bottom row) the asymptomatic plaque.

**Figure 3:**
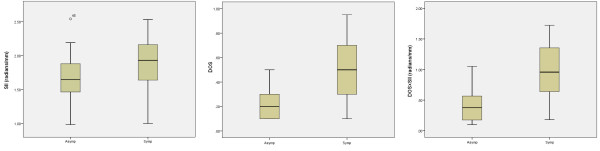
**Distribution of plaque SII (left), degrees of stenosis (DOS, middle) and the product of the two (right) in the symptomatic and asymptomatic plaque groups.** Degrees of stenosis are given as degree of stenosis (%)/100% (i.e. 0.5 corresponds to 50%, etc.).

**Figure 4:**
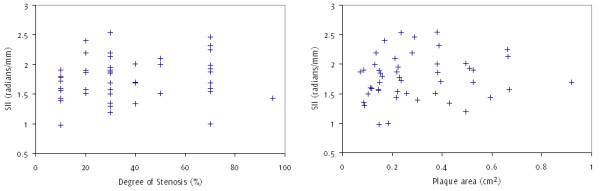
**Scatter plot of the plaque surface irregularity index *versus *** **the degree of stenosis of the corresponding artery (left) and the plaque area (right), illustrating a lack of association between these parameters.**

Examples of a symptomatic and an asymptomatic plaque, with their corresponding surface irregularity measurements, are shown in Figures 1 and 2. Across the full data-set, the mean SII of symptomatic plaques was 1.89 radians/mm compared with 1.67 radians/mm for the asymptomatic plaques. Plaque SII (p = 0.03), the degree of stenosis (p < 0.01), and the product of the two (p < 0.01) were all significantly higher in symptomatic plaques compared with the asymptomatic (Figure 3). There was no statistically significant relationship between the plaque surface irregularity index and the degree of stenosis or the plaque area (p = 0.30 for both, Figure 4).

**Figure 5:**
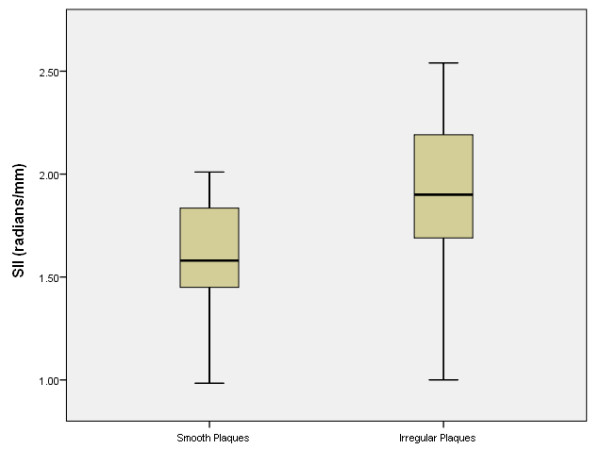
Distribution of plaque SII across the plaque groups qualitatively classified as having an irregular or smooth surface.

Qualitatively, 27 of the 47 plaques were classified as having an irregular surface, and 20 were classified as being smooth. Figure 1 illustrates examples of plaques qualitatively classified as having irregular and smooth surfaces. There were 11 smooth and 13 irregular plaques in the asymptomatic group, and 9 smooth and 14 irregular plaques in the symptomatic group. There was no statistically significant association between the qualitative assessment of surface irregularities and the symptomatic status (p = 0.64). However, the SII of the plaques qualitatively classified as having an irregular surface was significantly higher than those classified as having a smooth surface (p = 0.01, Figure 5).

**Figure 6:**
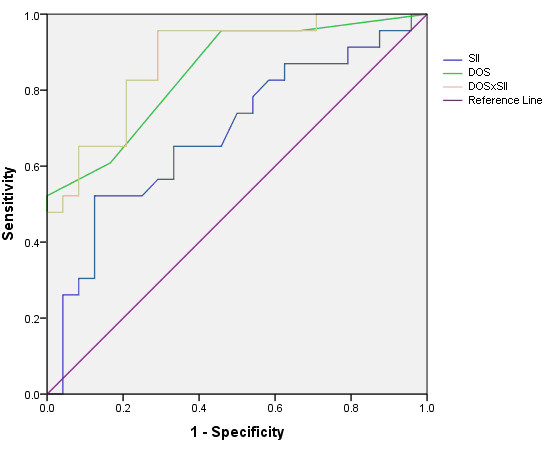
Comparison between ROC curves for plaque surface irregularity index (SII), the degree of stenosis (DOS) and their product (DOS × SII).

Receiver operating characteristic (ROC) curve analysis showed that the SII could predict the presence of ipsilateral hemispheric cerebrovascular symptoms with an accuracy of 66% (sensitivity 65%, specificity 67%) on its own and with an accuracy of 83% (sensitivity 96%, specificity 71%) in combination with the degree of stenosis (Figure 6). The area under the ROC curve was largest for the product of the degree of stenosis and the SII (0.866) compared to either the degree of stenosis (0.832) or the SII on its own (0.687).

Our study of plaque SII measurement reproducibility showed a mean intra-observer coefficient of variation of 4.4%. The mean intra-observer, inter-frame coefficient of variation was 10.6%.

## Discussion

This study defined a novel ultrasound plaque surface irregularity index which was found to have potential clinical value for improving the identification of the vulnerable carotid plaque. Ultrasound imaging provides a convenient and non-invasive means of assessing the carotid plaque. Of the characteristics of plaques that can be assessed using ultrasound, plaque surface structure is an interesting potential candidate for inclusion in a stroke risk model. However, there are two major practical problems with the ultrasound assessment of plaque surface structure. First, an irregular surface observed on ultrasound does not necessarily indicate an ulcerated or compromised plaque surface. Barry et al. [23], for example, found that false ultrasound diagnoses of ulceration could be due to culs-de-sac or pits in fibrotic tissue that look like ulcers. Secondly, ulcerations or surface defects may not always be detected, particularly in cross-sectional, 2-dimensional ultrasound imaging. This is due to the limited coverage of 2-dimensional ultrasound. Furthermore, small ulcerations or surface defects may not be revealed if these are smaller than the resolution of the ultrasound imaging system. Despite these difficulties, it is reasonable to expect potentially vulnerable types of plaque, such as plaques with ulcerations or plaques for which the surface integrity has been compromised, to exhibit greater irregularity in general. Irregular plaques could also potentially lead to more disturbed blood flow patterns with local high- and low-velocity flow regions and subsequent increases in plaque stress and increased risks of thrombosis, respectively. We should therefore expect an assessment of the surface irregularities of plaques to bring useful information that relates to plaque vulnerability. However, in a small cohort of patients, a strong correlation to symptoms should not be expected for the surface irregularities on their own, since it is an assessment only of the surfaces of plaques and surface irregularities may or may not be indicative of ulcerations and other surface defects.

In our study, we measured the surface irregularities of plaques in an objective manner and found that these quantitative measurements correlated with a qualitative assessment of surface irregularities. A correlation between surface irregularities and ipsilateral hemispheric symptoms was found for the novel quantitative method but not for the qualitative measure. The absence of a correlation in the case of the qualitative assessment can be attributed to the increased subjectivity of qualitative measures which may render a weak correlation to symptoms undetectable. The subjectivity of the qualitative assessment is most apparent with plaques that can not be classified as smooth or irregular with any certainty. In such cases, the assessor may make a highly subjective decision to place the plaque in one or the other group. The alternative is to mark such plaques as having an indeterminate surface characteristic and therefore unclassified.

We found that the combination of the plaque surface irregularity index with the degree of stenosis of the corresponding artery resulted in a more effective diagnostic test compared to the degree of stenosis on its own. This indicates that the objective study of plaque surface irregularities may provide useful additional information for predicting the presence of cerebrovascular symptoms. There was no significant correlation between the plaque SII and the degree of stenosis in our assessment, indicating that the former may provide information that is complementary to the latter.

Our surface irregularity index was combined with the degree of stenosis of the corresponding artery as the latter is an established parameter widely used in clinical practice and associated with an increased risk of cerebrovascular events. We took the product of the two parameters as the presence of ipsilateral hemispheric symptoms was directly related to both the degree of stenosis and the surface irregularity index. Our study found that combining the surface irregularity index with the degree of stenosis results in a more effective risk indicator than the degree of stenosis on its own.

The measurement technique we used had good reproducibility. The intra-observer variations were due to the human operator involvement required for the initial setup of the boundary detection procedure that resulted in the semi-automatic delineation of the plaque-arterial lumen boundaries, while the inter-frame variations were probably chiefly due to out-of-plane plaque, patient, and probe motion.

Further work can be directed towards studying the surface irregularities of plaques taking into account the echogenicity characteristics local to the surface. This would be useful as it may be more likely for surface irregularities to correspond to surface defects such as ulcerations or haemorrhages if the plaque has a less echogenic pattern (e.g. a ruptured fibrous cap or a haemorrhage) compared to being highly echogenic (e.g. fibrous or calcified). The variation of surface irregularities across plaque surfaces should also be explored in a follow-up study since plaque surfaces may contain both smooth and rough segments and their distribution may provide useful additional information that relates to plaque vulnerability.

## Conclusions

Our study has shown that an objective assessment of plaque surface irregularities using a novel surface irregularity index may correlate with the presence of ipsilateral hemispheric cerebrovascular symptoms. We found an increase in diagnostic performance with the use of the plaque SII versus that provided by the degree of stenosis alone. Plaque SII may therefore be a valuable tool for improving risk assessment, by means of helping identify the vulnerable plaques in patients with carotid artery disease. The potential clinical value of this parameter should be explored in follow-up studies.

## Competing interests

The authors declare that they have no competing interests.

## Authors’ contributions

The study was conceived by KVR and BK. Ultrasound data were collected by TCH. Algorithm development and analyses were carried out by BK. All authors contributed to the interpretation and presentation of the results and all authors read and approved the final manuscript.

## References

[B1] Leary DH Holen J Ricotta JJ Roe S Schenk EA Carotid bifurcation disease: prediction of ulceration with B-mode US Radiology 1987 162 523 525 3541034 10.1148/radiology.162.2.3541034

[B2] De Bray JM Baud JM Dauzat M Consensus Concerning the Morphology and the Risk of Carotid Plaques Cerebrovasc Dis 1997 7 289 296 10.1159/000108415

[B3] Muraki M Mikami T Yoshimoto T Fujimoto S Tokuda K Kaneko S New criteria for the sonographic diagnosis of a plaque ulcer in the extracranial carotid artery Am J Roentgenol 2012 198 1161 1166 10.2214/AJR.11.7018 22528908

[B4] Schminke U Motsch L Hilker L Kessler C Three-dimensional ultrasound observation of carotid artery plaque ulceration Stroke 2000 31 1651 1655 10.1161/01.STR.31.7.1651 10884468

[B5] Sitzer M Müller W Rademacher J Siebler M Hort W Kniemeyer HW Color-flow Doppler-assisted duplex imaging fails to detect ulceration in high-grade internal carotid artery stenosis J Vasc Surg 1996 23 461 465 10.1016/S0741-5214(96)80011-5 8601888

[B6] Steinke W Hennerici M Rautenberg W Mohr JP Symptomatic and asymptomatic high-grade carotid stenoses in Doppler color-flow imaging Neurology 1992 42 131 138 10.1212/WNL.42.1.131 1734294

[B7] Young N Soo YS Fischer P Comparison of duplex ultrasound with angiography in assessment of carotid bifurcation disease Australas Radiol 1992 36 54 58 10.1111/j.1440-1673.1992.tb03076.x 1632749

[B8] Aburahma AF Kyer PD Robinson PA Hannay RS The correlation of ultrasonic carotid plaque morphology and carotid plaque hemorrhage: clinical implications Surgery 1998 124 721 726 10.1067/msy.1998.91488 9780994

[B9] Aburahma AF Covelli MA Robinson PA Holt SM The role of carotid duplex ultrasound in evaluating plaque morphology: potential use in selecting patients for carotid stenting J Endovasc Surg 1999 6 59 65 10.1583/1074-6218(1999)006<0059:TROCDU>2.0.CO;2 10088891

[B10] Kessler C Von Maravic M Brückmann H Kömpf D Ultrasound for the assessment of the embolic risk of carotid plaques Acta Neurol Scand 1995 92 231 234 10.1111/j.1600-0447.1995.tb09574.x 7484077

[B11] Manolio TA Burke GL Leary DH Evans G Beauchamp N Knepper L Relationships of cerebral MRI findings to ultrasonographic carotid atherosclerosis in older adults : the Cardiovascular Health Study. CHS Collaborative Research Group Arterioscler Thromb Vasc Biol 1999 19 356 365 10.1161/01.ATV.19.2.356 9974419

[B12] Pedro LM Pedro MM Gonçalves I Carneiro TF Balsinha C Fernandes Fernandes R Computer-assisted carotid plaque analysis: characteristics of plaques associated with cerebrovascular symptoms and cerebral infarction Eur J Vasc Endovasc Surg 2000 19 118 123 10.1053/ejvs.1999.0952 10727359

[B13] Carra G Visonà A Bonanome A Lusiani L Pesavento R Bortolon M Carotid plaque morphology and cerebrovascular events Int Angiol 2003 22 284 289 14612856

[B14] Ding S Zhang M Zhao Y Chen W Yao G Zhang C The role of carotid plaque vulnerability and inflammation in the pathogenesis of acute ischemic stroke Am J Med Sci 2008 336 27 31 10.1097/MAJ.0b013e31815b60a1 18626232

[B15] Golledge J Cuming R Ellis M Davies AH Greenhalgh RM Carotid plaque characteristics and presenting symptom Br J Surg 1998 84 1697 1701 9448618

[B16] Meairs S Hennerici M Four-dimensional ultrasonographic characterization of plaque surface motion in patients with symptomatic and asymptomatic carotid artery stenosis Stroke 1999 30 1807 1813 10.1161/01.STR.30.9.1807 10471428

[B17] Denzel C Fellner F Wutke R Bazler K Müller K Lang W Ultrasonographic analysis of arteriosclerotic plaques in the internal carotid artery Eur J Ultrasound 2003 16 161 167 10.1016/S0929-8266(02)00069-1 12573784

[B18] Gaunt ME Brown L Hartshorne T Bell PR Naylor AR Unstable carotid plaques: preoperative identification and association with intraoperative embolisation detected by transcranial Doppler Eur J Vasc Endovasc Surg 1996 11 78 82 10.1016/S1078-5884(96)80139-0 8564492

[B19] Rubin JR Bondi JA Rhodes RS Duplex scanning versus conventional arteriography for the evaluation of carotid artery plaque morphology Surgery 1987 102 749 755 3310302

[B20] Van Damme H Vivario M Pathologic aspects of carotid plaques: surgical and clinical significance Int Angiol 1993 12 299 311 8207303

[B21] Widder B Paulat K Hackspacher J Hamann H Hutschenreiter S Kreutzer C Morphological characterization of carotid artery stenoses by ultrasound duplex scanning Ultrasound Med Biol 1990 16 349 354 10.1016/0301-5629(90)90064-J 2204161

[B22] Wolverson MK Bashiti HM Peterson GJ Ultrasonic tissue characterization of atheromatous plaques using a high resolution real time scanner Ultrasound Med Biol 1983 9 599 609 10.1016/0301-5629(83)90005-4 6670145

[B23] Barry R Pienaar C Nel CJ Accuracy of B-mode ultrasonography in detecting carotid plaque hemorrhage and ulceration Ann Vasc Surg 1990 4 466 470 10.1016/S0890-5096(07)60072-7 2223544

[B24] Bluth EI Mcvay LV Merritt CR Sullivan MA The identification of ulcerative plaque with high resolution duplex carotid scanning J Ultrasound Med 1988 7 73 76 3279227 10.7863/jum.1988.7.2.73

[B25] Comerota AJ Katz ML White JV Grosh JD The preoperative diagnosis of the ulcerated carotid atheroma J Vasc Surg 1990 11 505 510 2182913

[B26] European Carotid Plaque Study Group Carotid artery plaque composition–relationship to clinical presentation and ultrasound B-mode imaging Eur J Vasc Endovasc Surg 1995 10 23 30 7633965 10.1016/s1078-5884(05)80194-7

[B27] Prabhakaran S Rundek T Ramas R Elkind MSV Paik MC Boden-albala B Carotid plaque surface irregularity predicts ischemic stroke: the northern Manhattan study Stroke 2006 37 2696 2701 10.1161/01.STR.0000244780.82190.a4 17008627 PMC2654324

[B28] Tegos TJ Kalomiris KJ Sabetai MM Kalodiki E Nicolaides AN Significance of sonographic tissue and surface characteristics of carotid plaques Am J Neuroradiol 2001 22 1605 1612 11559516 PMC7974562

[B29] Chiu B Beletsky V Spence JD Parraga G Fenster A Analysis of carotid lumen surface morphology using three-dimensional ultrasound imaging Phys Med Biol 2009 54 1149 1167 10.1088/0031-9155/54/5/004 19174598

[B30] Fenster A Blake C Gyacskov I Landry A Spence JD 3D ultrasound analysis of carotid plaque volume and surface morphology Ultrasonics 2006 44 E153 E157 16844159 10.1016/j.ultras.2006.06.027

[B31] Kanber B Hartshorne TC Horsfield MA Naylor AR Robinson TG Ramnarine KV Dynamic variations in the ultrasound greyscale median of carotid artery plaques Cardiovasc Ultrasound 2013 11 21 10.1186/1476-7120-11-21 23767988 PMC3686622

[B32] North American Symptomatic Carotid Endarterectomy Trial Collaborators Beneficial effect of carotid endarterectomy in symptomatic patients with high-grade carotid stenosis N Engl J Med 1991 325 445 453 1852179 10.1056/NEJM199108153250701

[B33] Grant EG Benson CB Moneta GL Alexandrov AV Baker JD Bluth EI Carotid artery stenosis: gray-scale and Doppler US diagnosis–Society of Radiologists in Ultrasound Consensus Conference Radiology 2003 229 340 346 10.1148/radiol.2292030516 14500855

[B34] Oates CP Naylor AR Hartshorne T Charles SM Fail T Humphries K Joint recommendations for reporting carotid ultrasound investigations in the United Kingdom Eur J Vasc Endovasc Surg 2008 37 251 261 19046904 10.1016/j.ejvs.2008.10.015

